# Dose-response effect of berberine on bile acid profile and gut microbiota in mice

**DOI:** 10.1186/s12906-016-1367-7

**Published:** 2016-10-18

**Authors:** Ying Guo, YouCai Zhang, WeiHua Huang, Felcy Pavithra Selwyn, Curtis D. Klaassen

**Affiliations:** 1 Department of Clinical Pharmacology, Xiangya Hospital, Central South University, Changsha, 410008 People’s Republic of China; 2 University of Kansas Medical Center, Kansas City, KS 66160 USA; 3 Institute of Clinical Pharmacology, Hunan Key Laboratory of Pharmacogenetics, Central South University, Changsha, 410078 People’s Republic of China; 4 2617 W 112th Street, Leawood, KS 66211 USA

**Keywords:** Berberine, Bile acids, Gut Microbiota, Mice

## Abstract

**Background:**

Berberine (BBR) is a traditional antimicrobial herbal medicine. Recently, BBR has gained popularity as a supplement to lower blood lipids, cholesterol and glucose. Bile acids (BAs) are known to regulate blood levels of triglycerides, cholesterol, glucose and energy homeostasis, and gut flora play an important role in BA metabolism. However, whether BBR alters BAs metabolism or dose-response effect of BBR on gut flora is unknown.

**Methods:**

In this study, the effects of various doses of BBR on the concentrations of BAs in liver and serum of male C57BL/6 mice were determined by UPLC-MS/MS, and the expression of BA-related genes, as well as the amount of 32 of the most abundant gut bacterial species in the terminal ileum and large intestine of male C57BL/6 mice were quantified by RT-PCR and Quantigene 2.0 Reagent System, respectively.

**Results:**

Unconjugated BAs and total BAs were significantly altered by BBR in serum but not in liver. Increased primary BAs (βMCA, TβMCA and TUDCA) and decreased secondary BAs (DCA, LCA and the T-conjugates) were observed in livers and serum of mice fed BBR. The expression of BA-synthetic enzymes (Cyp7a1 and 8b1) and uptake transporter (Ntcp) increased 39-400 % in liver of mice fed the higher doses of BBR, whereas nuclear receptors and efflux transporters were not markedly altered. In addition, *Bacteroides* were enriched in the terminal ileum and large bowel of mice treated with BBR.

**Conclusion:**

The present study indicated that various doses of BBR have effects on BA metabolism and related genes as well as intestinal flora, which provides insight into many pathways of BBR effects.

**Electronic supplementary material:**

The online version of this article (doi:10.1186/s12906-016-1367-7) contains supplementary material, which is available to authorized users.

## Background

Bile acids (BAs) are important natural detergents that form micelles to facilitate the absorption of dietary fat and lipid soluble vitamins from the gastrointestinal tract. BAs are also the driving force for bile formation, and can reduce bacteria in the biliary tract and intestine [1]. In addition, BAs are important metabolic and inflammatory signaling molecules as they regulate lipid- and energy-related nuclear hormone receptors, such as farnesoid-X-receptor (FXR) and transmembrane G-protein-coupled receptor 5 (TGR5 or GPBAR1) [2, 3].

Primary BAs are synthesized in the liver and are converted to secondary BAs by bacteria in the intestine. Secondary BAs are more toxic than primary BAs, possibly because of their higher hydrophobicity [4]. For instance, deoxycholic acid (DCA) and lithocholic acid (LCA), which are secondary BAs produced by bacteria, are thought to play roles in colorectal cancer, liver cancer, and cholesterol gallstones [5–8].

BAs are synthesized and circulate in the liver and intestine, which is orchestrated by feedback loops [6]. The liver synthesizes primary BAs, namely cholic acid (CA) and chenodeoxycholic acid (CDCA) from cholesterol. In rodents, CDCA is further hydroxylated to alpha-muricholic acid (αMCA), and then epimerized to beta-muricholic acid (βMCA). The rate-limiting enzyme of primary BA synthesis is Cytochrome P450 (Cyp) 7a1 (cholesterol 7α-hydroxylase), which initiates the classic synthetic pathway, whereas Cyp27a1 and Cyp7b1 are important in the alternative route of BA synthesis [9]. Primary BAs are conjugated with glycine (predominant in human) or taurine (predominant in rodent) in the liver. A portion of the BAs are directly effluxed into the blood by the efflux transporters on the basolateral membrane of hepatocytes, including multiple drug resistance-related protein (Mrp) 3, Mrp4 and organic solute transporter (Ost) β. However, most BAs in the liver are pumped into the biliary tree by the efflux transporters on the canalicular membrane of hepatocytes, mainly the bile salt export pump (Bsep). The BAs are stored in the gallbladder to be further concentrated [10], and thereafter delivered into the intestine to promote emulsification of lipids and fat soluble vitamins [11]. In the intestine, primary BAs are deconjugated, dehydroxylated at C-7, epimerized, and oxidized to form secondary BAs in the terminal ileum and large bowel by anaerobic bacteria [12]. In the ileum, through a BA-activated FXR-dependent pathway, fibroblast growth factor 15 (FGF15) is secreted into the blood, and interacts with the fibroblast growth factor receptor 4 (Fgfr4) in the hepatocytes to inhibit BA synthesis [13, 14]. In the liver, BAs activate FXR, which induce the expression of small heterodimer partner (SHP) that represses liver receptor homolog-1 (LRH-1), leading to decreased transcription of Cyp7a1 [15]. Thus BAs regulate their own synthesis by FXR-Fgf15 in the intestine and FXR-SHP in the liver. CDCA, TCDCA, TCA, TDCA, DCA and CA are potent activators of FXR activity [16, 17], whereas TβMCA and UDCA are natural antagonists of FXR [14, 18]. However, the entire regulatory mechanism of BA synthesis is not fully understood.

**Fig. 1:**
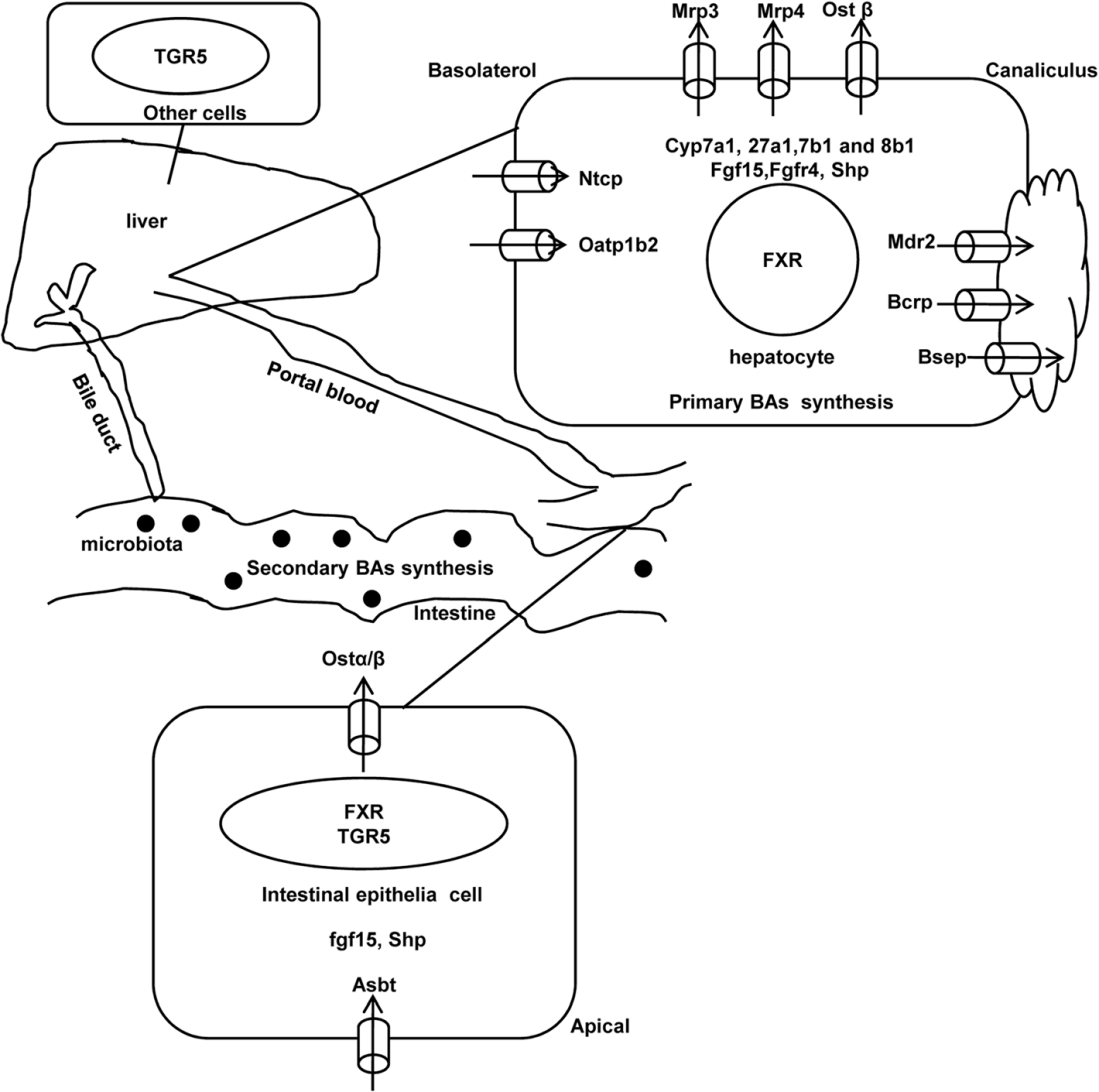
Schematic diagram of BAs EHC. This is the recirculation of BAs between the intestine and the liver. BAs are synthesized in the hepatocyte (from cholesterol) by Cyp7a1, 27a1, 7b1 and 8b1, released into bile, transformed by the gut microbiota, reabsorbed in the small intestine, and returned to the liver to be recycled. The major genes involved in the EHC are shown

About 5 % of secreted BAs in the gut are excreted into feces and is the most important eliminating channel of cholesterol from the body [19], whereas, the remaining 95 % are reclaimed at the terminal ileum by the apical sodium-dependent BA uptake transporter (Asbt) into the ileocytes, followed by Ostα/β active transport to the portal blood. The BAs are then taken up into hepatocytes by the sodium taurocholate cotransporting polypeptide (Ntcp: uptake of conjugated BAs) and the organic anion transporting peptide 1b2 (Oatp1b2: uptake of unconjugated BAs) [20]. This entire process is termed the enterohepatic circulation (EHC) [10] (Fig. 1).

Berberine (BBR) is a quaternary ammonium salt from the protoberberine group of isoquinoline alkaloids, and it is traditionally used for gastrointestinal inflammation in Chinese and native American medicines [21, 22]. Recently, because its potential cytostatic, antiproliferative, and antioxidative activities, the raw herb of BBR was ranked as the sixth most commonly used herbal supplement for children in America [23]. BBR has been reported to have inhibitory effects against *Staphylococcus aureus*, *Plasmodium falciparum*, cholera, amoebiasis, and viruses, but also affects the gut microflora including *Bifidobacterium longum*, *Bifidobacterium bifidum*, *Clostridium perfringens*, and *Clostridium paraputrificum* [22, 24, 25]. It has been reported that modulation of the gut microbiota by BBR may contribute to its antidiabetic effect [26, 27]. BBR is also becoming widely used as a supplement to prevent hypercholesterolemia for decreasing cholesterol absorption from the intestine and stimulating BA synthesis [28, 29].

Antibiotics affect BA metabolism theoretically due to their ability to alter intestinal bacteria, which play a fundamental role not only on the generation of secondary BAs, but also as a modulator of hepatic BA synthesis [14, 30]. BBR is also proven to stimulate bile secretion [31, 32], however, it is not clear if and how BBR affects BA concentrations, transporters involved in the EHC of BAs, and the abundance of individual gut microbiota.

To systematically explore the impact of different doses of BBR on BA profiles in liver and serum and the potential mechanism for these alterations, in the present study, BBR were given to mice, and concentrations of total BAs, individual BAs, and genes involved in BA homeostasis, as well as bacteria in the terminal ileum and large intestine were quantified. Various doses of BBR increased primary BAs, whereas it decreased secondary BAs, and has effects on BA metabolism and related genes as well as intestinal flora, which provides insight into many pathways of BBR effects.

## Methods

### Ethics statement

Mice were housed according to guidelines of the Institutional Animal Care and Use Committee at the University of Kansas Medical Center. Procedures were carried out in compliance with standards for the use of laboratory animals. Animal experiments performed in this manuscript were approved by the Institutional Animal Care and Use Committee at the University of Kansas Medical Center.

### Animals and treatments

Seven-week-old male C57BL/6 mice were purchased from Charles River Laboratories, Inc. (Wilmington, MA), housed according to the American Animal Association Laboratory Animal Care guidance under a standard 12-h dark-light cycle and humidity-controlled environment with a room temperature at approximately 25 °C, and acclimated for at least 1 week before treatment. Mice were arbitrarily divided into six groups and had access to Laboratory Rodent Chow 8604 (Harlan, Madison, WI) and drinking water *ad libitum*.

### Sample collection

BBR (B3251) was purchased from Sigma-Aldrich (USA). Six doses (0, 3, 10, 30, 100, 300 mg/kg) of BBR were given to mice by gavage (4-6 for each group) for 2 weeks after a pilot study. Control animals received the vehicle (saline; 0 mg/kg) only. Mice were anesthetized, blood was collected by orbital bleeding, and serum was obtained by centrifuging blood at 6,000 g for 15 min. Livers with gallbladders removed were harvested from the same animals, washed, frozen in liquid nitrogen, and stored at -80 °C. Ileum and large intestine were separated and the contents of the terminal ileum and large bowel were collected into 3 ml of phosphate buffered saline containing 10 mM dithiothreitol (DTT). All tissues and contents were stored at -80 °C until use.

### UPLC-MS/MS analysis of BAs

Serum and liver samples were prepared and analyzed as described previously [33] with modification [11]. Individual bile acids were quantified by ultraperformance liquid chromatography–mass spectrometry (UPLC-MS/MS) according to peak areas and a series of working standard curves as described previously [11, 33]. The standards included unconjugated bile acids, which are cholic acid (CA), chenodeoxycholic acid (CDCA), α, β and ω muricholic acids (MCA), deoxycholic acid (DCA), lithocholic acid (LCA), ursodeoxycholic acid (UDCA), hyodeoxycholic acid (HDCA), and murideoxycholic acid (MDCA), as well as taurine (T) conjugates, which are TCA, TCDCA, TDCA, TLCA, TUDCA, and TMCA. Quantification of TωMCA and ωMCA were relative to TαMCA and αMCA respectively.

### Total RNA isolation

Approximately 50 mg of liver or ileum were homogenized in 1 ml RNAzol Bee reagent (Tel-Test Inc., Friendswood, TX). Total messenger RNA (mRNA) was isolated according to the manufacturer’s protocol, and concentrations were quantified using a NanoDrop Spectrophotometer (NanoDrop Technologies, Wilmington, DE) at 260 nm. Formaldehyde-agarose gel eletrophoresis was used for evaluating the integrity of these total RNA samples, which were confirmed by visualization of the 18 s and 28 s rRNA bands. The diethyl pyrocarbamate (DEPC)-treated double-distilled water was used to dilute each of the RNA samples to 50 ng/μl for real-time PCR quantification.

### Synthesis of cDNA and real-time PCR quantification

Reverse transcription of RNA to cDNA was performed using the Applied Biosystems High Capacity Reverse Transcriptase kit (Applied Biosystems, Foster City, CA). Subsequently, quantitative PCR was performed on the resulting cDNA using SYBR Green PCR Master Mix in 7300HT Fast Real-Time PCR System (Applied Biosystems). Primers (Additional file 1: Table S1) for RT-PCR were synthesized by Integrated DNA Technologies (Coralville, IA).

### Bacterial DNA extraction and quantification

Bacterial DNA was extracted by QIAmp DNA® stool kit (Qiagen, Valencia, CA) following the instructions**,** and then techniques that rely on 16S rRNA gene sequences were used for the identification and classification of bacterial species as described previously [30, 34]. In brief, intestinal contents were centrifuged at 20,000 g for 30 min at 4 °C. Total genomic bacterial DNA was extracted from the pellet. The integrity, concentration, and quality of the total DNA were assessed by agarose gel electrophoresis, and determined by absorption at A_260_, and A_260_ to A_280_ ratio, respectively. The 16S rDNA gene of 32 bacteria was quantified in pooled and individual samples using Quantigene 2.0 Reagent System (Panomics/Affymetrix, Fremont, CA) according to the manufacturer’s protocol.

### Statistical analysis

Data are expressed as mean ± S.E. (*n* = 4-6). Differences between mean values were tested for statistical significance (*P* < 0.05) by one-way analysis of variance (ANOVA) followed by Duncan’s *post hoc* test. Spearman’s rank test was conducted to analyze the associations between BBR concentrations and BA profile, related genes and gut microbiota in mice (SPSS Inc., Chicago, IL, USA, version 16.0). Statistical significance was set at *P* < 0 · 05 for all analyses.

## Results

### Concentrations of T-conjugated BAs, unconjugated BAs and total BAs in livers and serum of control and BBR-treated mice

**Fig. 2:**
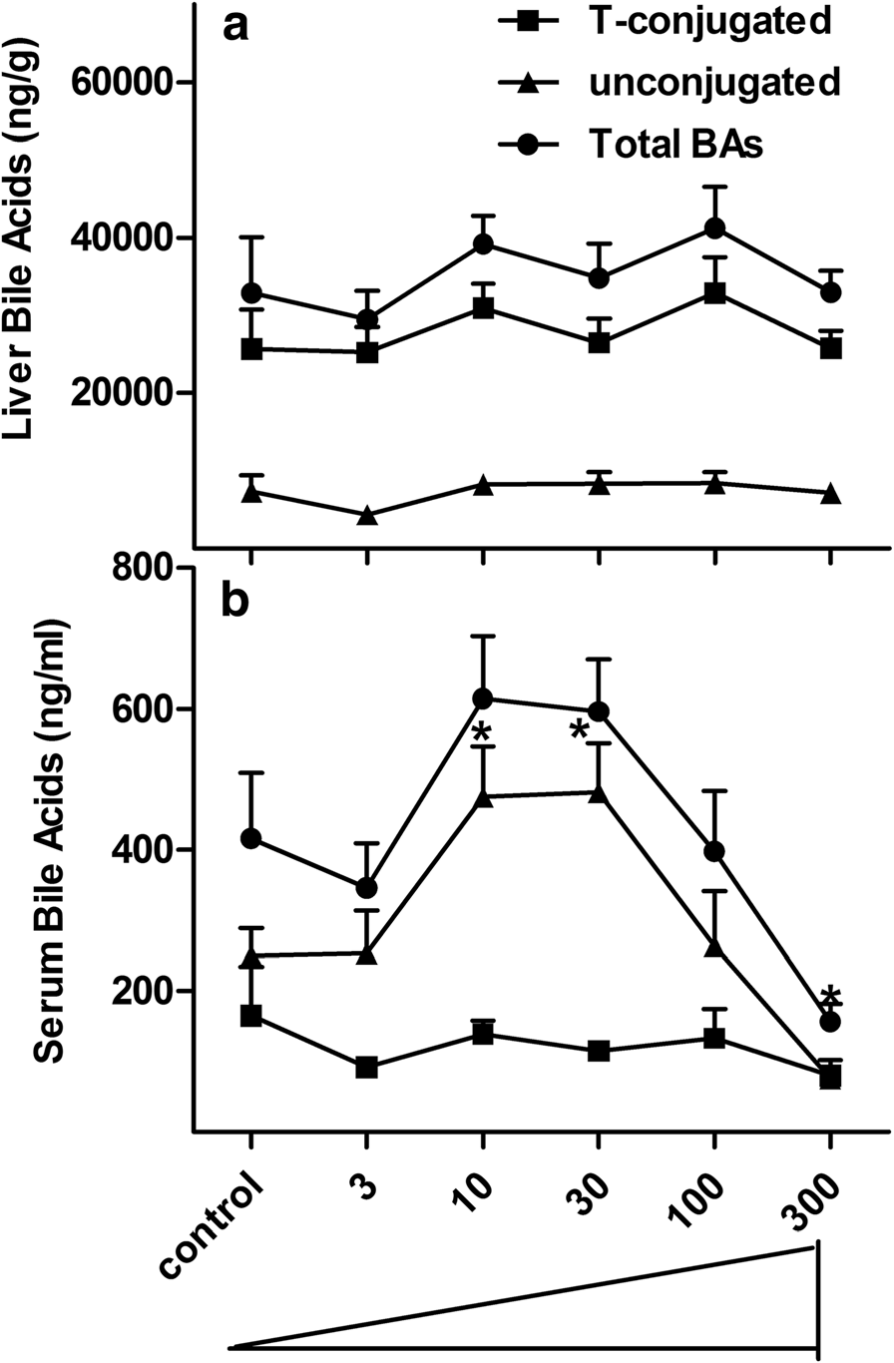
Concentrations of T-conjugated BAs, unconjugated BAs and total BAs in control and BBR-treated mice. T-conjugated, unconjugated BAs and total BAs in liver and serum were quantified and the amounts are showed in **a** and **b**, respectively. All values are expressed as mean ± S.E.M. ANOVA followed by Duncan’s post hoc test were used, and * means *P* < 0.05 when compared with controls

The T-conjugated BAs and total BAs did not change significantly in livers of the BBR-treated mice (Fig. 2a), however, in serum, the unconjugated BAs significantly increased (*P* = 0.0017) about 90–92 % at the two middle doses of BBR (10 and 30 mg/kg) while they tended to decrease after the highest dose. The total BAs in serum had a similar trend to unconjugated BAs, with a significant decrease (*P* = 0.006, 62 %) after the highest dose of BBR. There was no significant change in the T-conjugated BAs in serum of mice fed the various doses of BBR (Fig. 2b).

### Concentrations of individual BAs in livers of control and BBR-treated mice

**Fig. 3:**
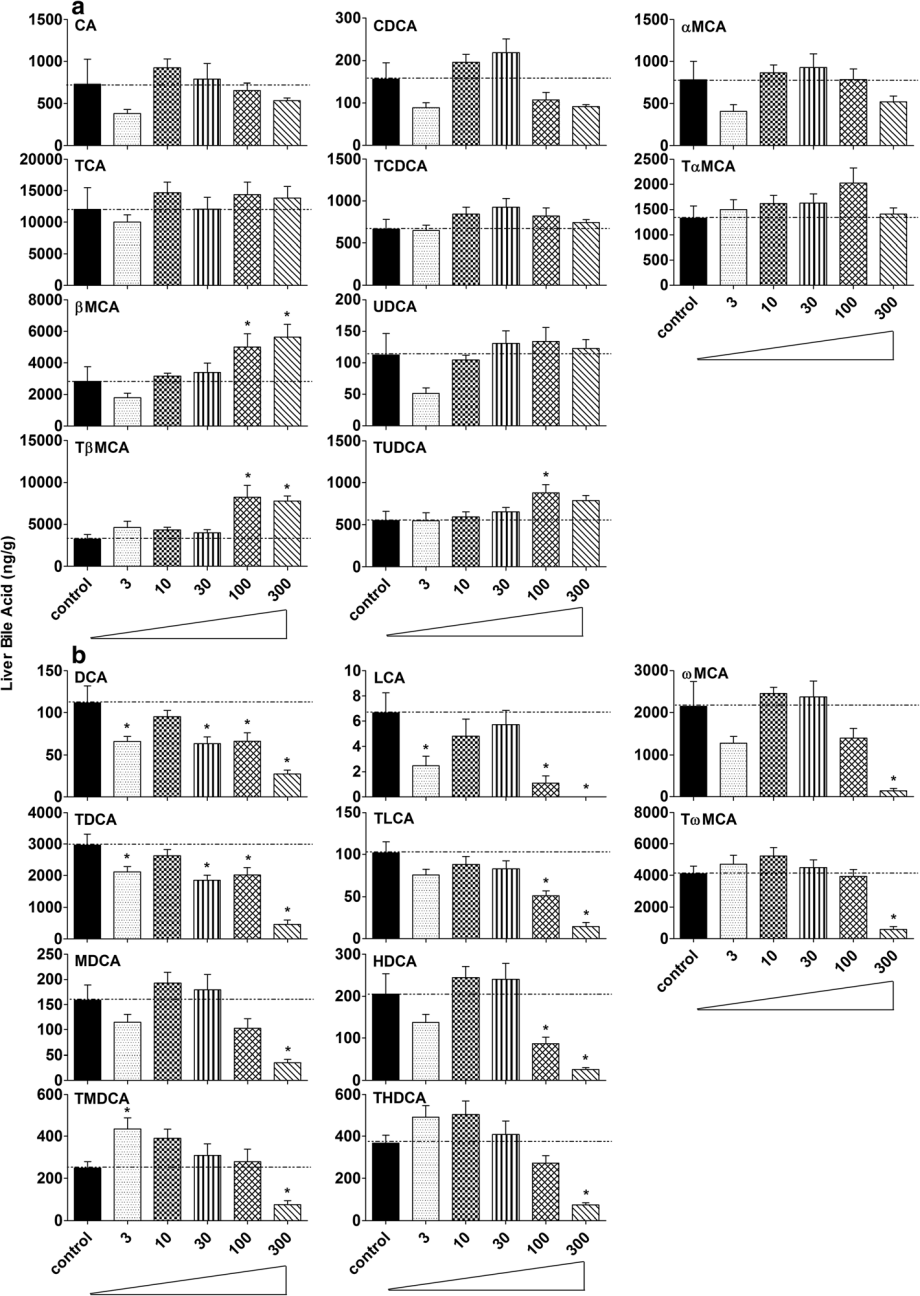
Concentrations of individual BAs in livers of control and BBR-treated mice. Twenty individual BAs were quantified in livers of 8-week-old control and BBR-treated mice. **a** showed the amounts of 10 primary BAs, and **b** showed the amount of 10 secondary BAs. All values are expressed as mean ± S.E.M. ANOVA followed by Duncan’s post hoc test were used, and * means *P* < 0.05 when compared with controls

Twenty individual BAs were quantified in livers of control and BBR-treated mice (Fig. 3a–b). Of the 10 primary BAs, βMCA (*P* = 0.004) and TβMCA (*P* = 0.001) were increased about 100 % in mice treated with 100 or 300 mg/kg BBR, and TUDCA was increased 60 % in livers of mice treated with 100 mg/kg BBR (*P* = 0.038), whereas the other BAs were not significantly changed (Fig. 3a). BBR significantly decreased the secondary BAs (DCA: *P* = 0.000006; TDCA: *P* = 0.000002; LCA: *P* = 0.0009; ωMCA: *P* = 0.00027; TωMCA: *P* = 0.00004; MDCA: *P* = 0.001; TMDCA: *P* = 0.001; HDCA: *P* = 0.00000003; THDCA: *P* = 0.000000002. Fig. 3b). BBR at the highest dose (300 mg/kg) decreased the concentration of all the secondary BAs in liver. The second to the highest dose of BBR (100 mg/kg) decreased DCA, TDCA, LCA, TLCA, and HDCA. Lower doses of BBR also decreased DCA and TDCA.

### Concentrations of individual BAs in serum of control and BBR-treated mice

**Fig. 4:**
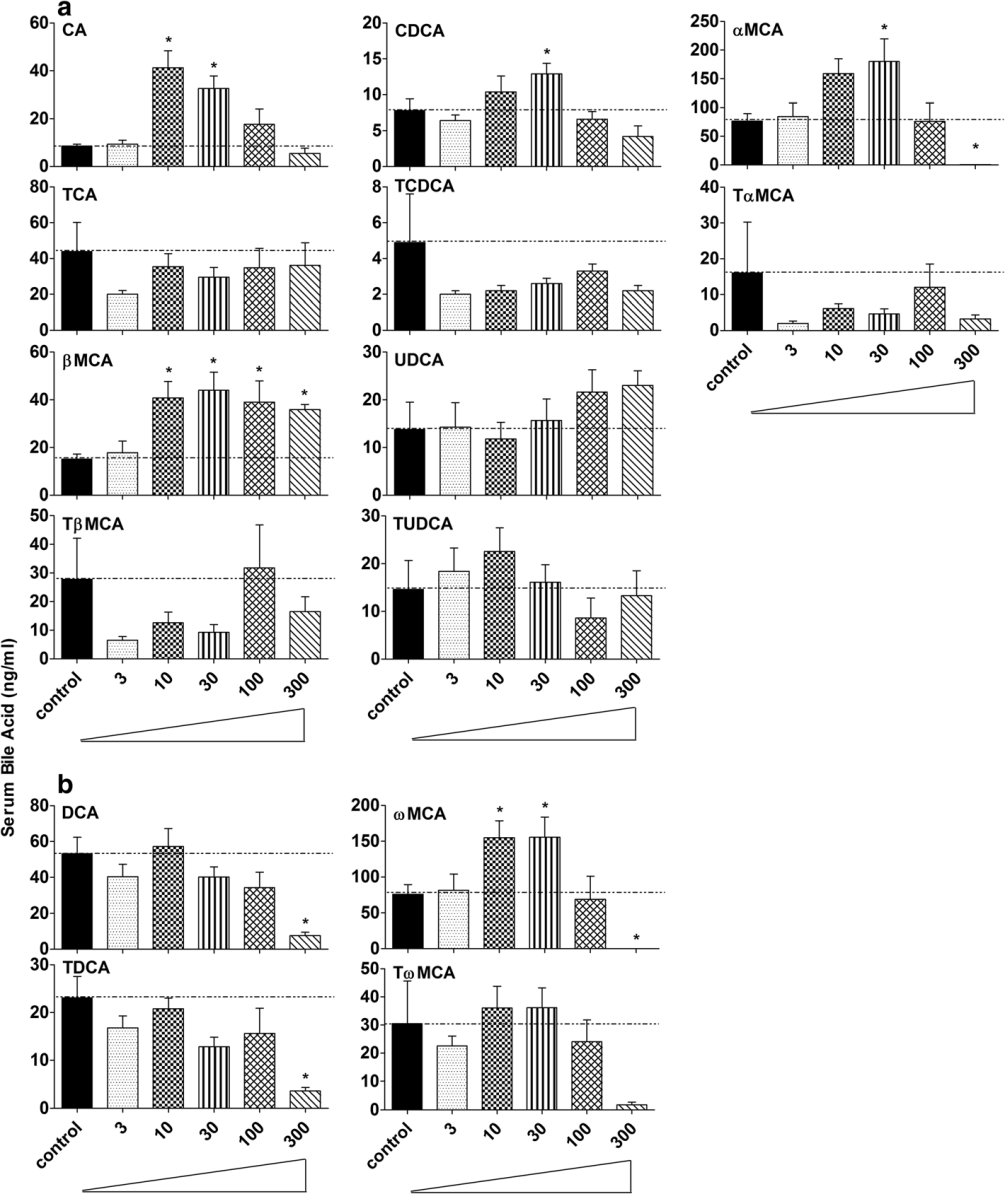
Concentrations of individual BAs in serum of control and BBR-treated mice. Eight primary (**a**) and four secondary BAs (**b**) were detected in serum of control and BBR-treated mice. All values are expressed as mean ± S.E.M. ANOVA followed by Duncan’s post hoc test were used, and * means *P* < 0.05 when compared with controls

Eight primary and four secondary BAs were detected in serum of control and BBR-treated mice (Fig. 4a–b). BA concentrations in serum were much lower than that in liver. Coincident with the rise of primary BAs in liver, βMCA in serum was about 100 % higher (*P* = 0.012) in all the BBR-treated mice except for the lowest dose (Fig. 4a). Moreover, BBR increased CA 300–400 % in the 10 and 30 mg/kg groups (*P* = 0.00006), and the 30 mg/kg increased CDCA (*P* = 0.007) about 50 %. BBR at 30 mg/kg increased αMCA about 300 %, however, no αMCA was detected in the serum after 300 mg/kg (*P* = 0.003). The secondary BAs in serum, including DCA (*P* = 0.007) and TDCA (*P* = 0.016), decreased about 85 % after the highest dose of BBR (Fig. 4b). The changes of ωMCA in serum was similar to that of αMCA, that is the intermediate doses of BBR doubled their concentrations (*P* = 0.002) in serum.

### mRNA expression of major BA-related genes in livers of control and BBR-treated mice

**Fig. 5:**
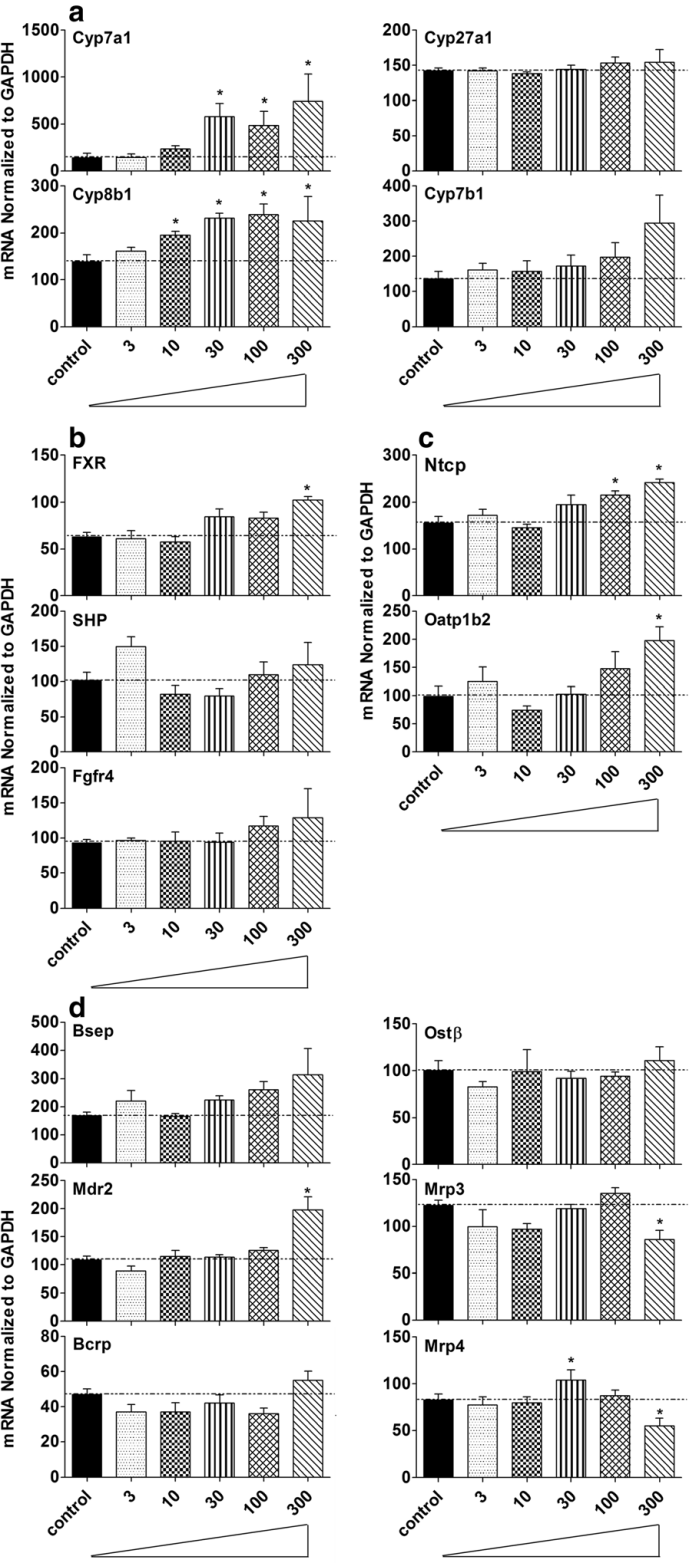
mRNA expression of major BA-related genes in livers of control and BBR-treated mice were examined (Fig. 5). BA synthetic enzymes involved in classical and alternative pathway were quantified in liver of BBR-treated mice (**a**). The mRNA expression of major BAs regulating factors in liver including FXR, SHP and Fgfr4 is demonstrated in (**b**). **c** illustrates the mRNA expression of major uptake transporters on the basolateral membrane of hepatocyte, which include Ntcp, Oatp1b2. The mRNA expression of major efflux transporters on the basolateral and canalicular membrane of hepatocyte are shown in (**d**). The left panel in **d** demonstrates the alteration of major efflux transporters on the canalicular membrane. The right panel of **d** shows the change of major efflux transporters on the basolateral membrane transporting the bile acids back to the blood after various doses of BBR. All values are expressed as mean ± S.E.M. ANOVA followed by Duncan’s post hoc test were used, and * means *P* < 0.05 when compared with controls

To investigate probable mechanisms for the changes of BA concentrations in mice treated with BBR, major genes that are involved in BA synthesis, uptake, efflux and regulation of the processes were examined (Fig. 5).

Enzymes involved in the classical and alternative pathways of BA synthesis were quantified in livers of BBR-treated mice (Fig. 5a). After BBR treatment, the mRNA of Cyp7a1, which is the rate-limiting enzyme in the classic pathway increased 200–400 % (*P* = 0.001), and Cyp8b1 mRNA increased up to 100 % (*P* = 0.003). The other enzymes involved in BA synthesis had a relatively minor change.

The mRNA expression of major factors regulating BAs in liver, including FXR, SHP and Fgfr4 are shown in Fig. 5b. Induction of FXR was less than 50 % in liver of mice treated with 300 mg/kg BBR (*P* = 0.001), and there was no significant difference of SHP or Fgfr4 mRNA expression in mice fed BBR.

Figure 5c illustrates the mRNA expression of major uptake transporters on the basolateral membrane of hepatocytes, which includes the Na^+^-dependent taurocholate cotransport peptide (Ntcp), and the organic anion–transporting polypeptide (Oatp) 1b2. Ntcp increased less than 50 % (*P* = 0.0003), and Oatp1b2 was increased about 100 % after 100 and 300 mg/kg BBR (*P* = 0.013).

The mRNA expression of major efflux transporters on the basolateral and canalicular membranes of hepatocytes is shown in Fig. 5d. The left panel of Fig. 5d demonstrates the alteration of major efflux transporters on the canalicular membrane, which transport chemicals into bile. There were no significant changes for most of these transporters after BBR treatment, however, Mdr2 was increased about 80 % after the highest dose of BBR (*P* = 0.000007). The right panel of Fig. 5d shows the major efflux transporters on the basolateral membrane that transport chemicals from the liver back to the blood. Mrp3 (*P* = 0.018) and Mrp4 (*P* = 0.020) were decreased about 30 % in mice that received the highest dose of BBR.

### mRNA expression of major regulating factors and transporters in ileum of control and BBR-treated mice

**Fig. 6:**
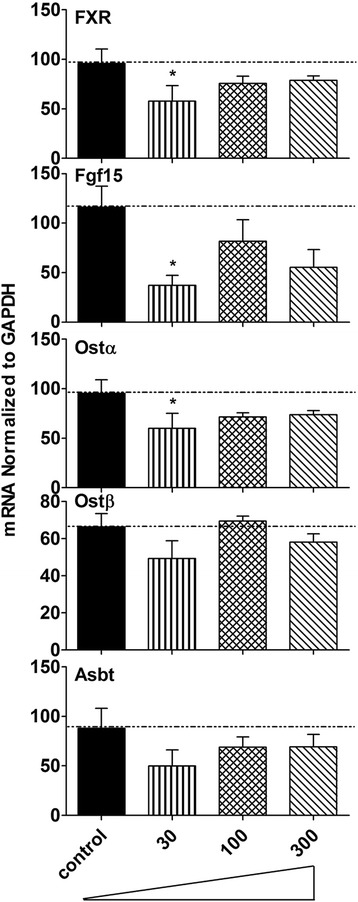
mRNA expression of major regulating factors and transporters including FXR, Fgf15, Ostα, Ostβ and Asbt in ileum of control and BBR-treated mice were quantified with the 3 higher doses groups. All values are expressed as mean ± S.E.M. ANOVA followed by Duncan’s post hoc test were used, and * means *P* < 0.05 when compared with controls

Because BA concentrations only changed in mice that were given doses of BBR above 30 mg/kg, mRNA expression of FXR, Fgf15, Ostα, Ostβ and Asbt were quantified in the mice given the 3 highest doses of BBR (Fig. 6). Although not statistically significant, mRNA expression of these BA related genes after 100 and 300 mg/kg BBR tended to decrease. However, mice administered 30 mg/kg BBR had decreased expression of FXR and Ostα (37–39 % suppression) and Fgf15 (68 % suppression) in ileum (*P* = 0.05).

### Bacteria in terminal ileum and large intestinal contents of control and BBR-treated mice

**Fig. 7:**
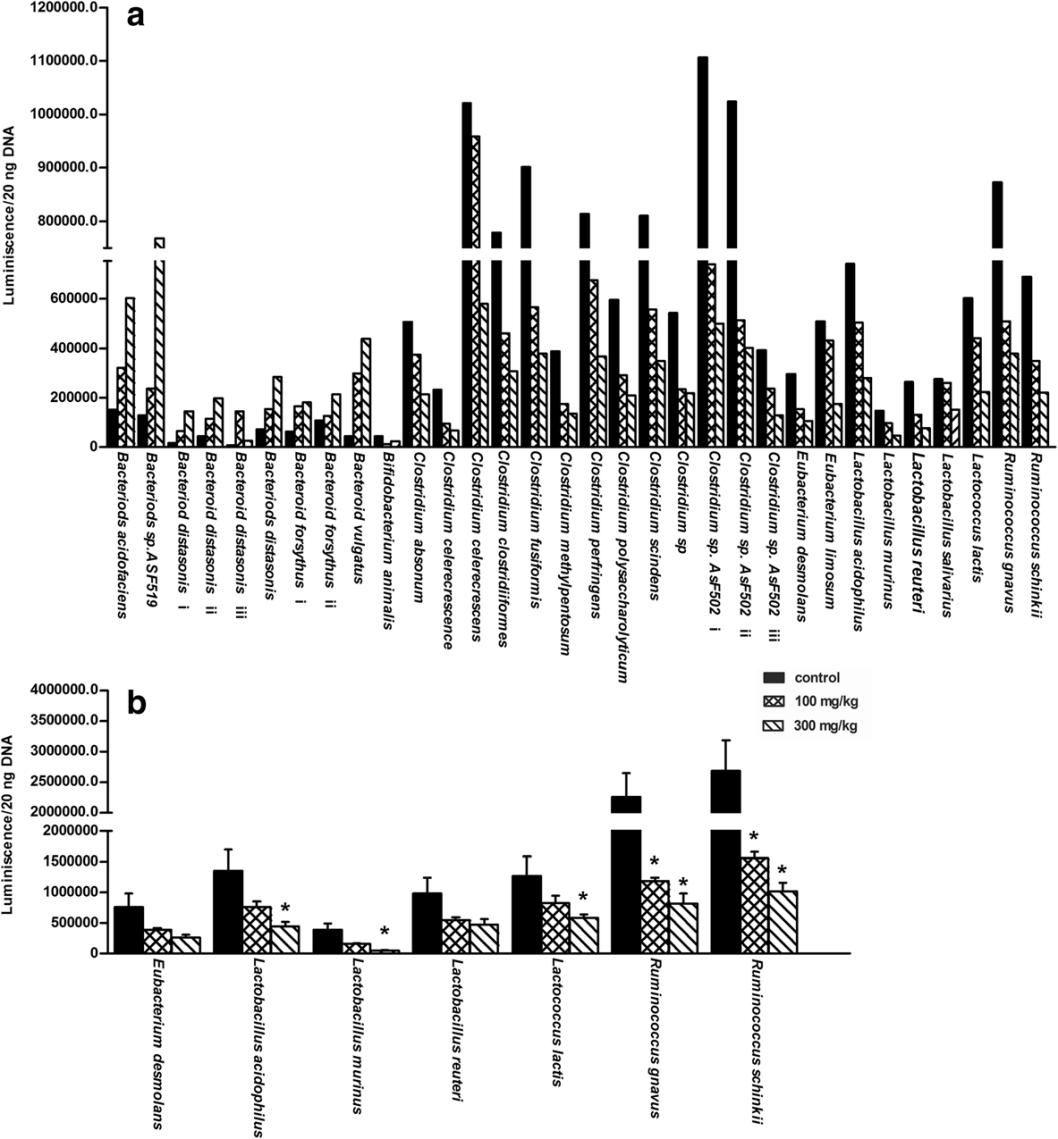
Bacteria in terminal ileum and large intestinal contents of control and BBR-treated mice. The relative amounts of 32 of the most abundant gut bacterial species in mice were quantified in the pooled samples of control, 100 and 300 mg/kg BBR treated groups (**a**), and validated by 7 bacteria in individual samples (**b**). All values are expressed as mean ± S.E.M. ANOVA followed by Duncan’s post hoc test were used, and * means *P* < 0.05 when compared with controls

BBR-induced decreases in the concentration of secondary BAs were most evident in mice after the two highest doses of BBR, therefore, the relative amounts of 16S rRNA for 32 of the most abundant gut bacterial species in mice were quantified in pooled samples of control, 100 and 300 mg/kg BBR-treated groups (Fig. 7a), and validated by 7 bacteria in individual samples (Fig. 7b). In pooled samples, *Bacteroides* increased, but other bacteria decreased with the increasing dosage of BBR. In the individual samples, *Ruminococcus gnavus* and *Ruminococcus schinkii* decreased about 40 % (*P* = 0.007) and 60 % (*P* = 0.011) in the 100 and 300 mg/kg BBR-treated groups, respectively. *Lactobacillus acidophilus*, *Lactobacillus murinus* and *Lactococcus lactis* decreased about 60 to 90 % in the 300 mg/kg BBR treated mice (*Lactobacillus acidophilus*: *P* = 0.013; *Lactobacillus murinus*: *P* = 0.002; *Lactococcus lactis*: *P* = 0.05). The changes were similar in the pooled versus the individual samples.

### Dose-response of BBR in mice

The associations between BBR concentrations and BA profile, related genes and gut microbiota in mice were analyzed, and the *P* (those are smaller than 0.05) and R values are shown in Table 1. The primary BAs in livers and serum, as well as related genes in the livers of BBR-treated mice including Oatp1b2, Bsep, Mdr2, Cyp8b1, Cyp7a1, Ntcp and FXR (in liver) were positively correlated to the increase of BBR concentrations, whereas secondary BAs and bacteria including *Ruminococcus gnavus, Ruminococcus schinkii, Lactobacillus acidophilus*, *Lactobacillus murinus* and *Lactococcus lactis* in terminal ileum and large intestinal contents were negatively correlated to the increase of BBR concentrations.

**Table 1:** The associations between BBR concentrations and BA, related genes and gut microbiota in mice

Name	Spearman R value	*P* value (<0.05)
Bile acids in the liver		
TLCA	−0.7140	0.000003
TDCA	−0.6321	0.00008
THDCA	−0.5581	0.0007
DCA	−0.5574	0.0008
LCA	−0.5341	0.001
HDCA	−0.4834	0.004
TMDCA	−0.4045	0.02
MDCA	−0.3991	0.021
TωMCA	−0.3918	0.024
UDCA	0.4347	0.011
TUDCA	0.5034	0.003
TβMCA	0.5898	0.0003
βMCA	0.6153	0.0001
Bile acids in the serum		
TDCA	−0.5305	0.001
DCA	−0.5062	0.003
βMCA	0.5112	0.002
Bile acids-related genes		
Oatp1b2	0.4137	0.017
Bsep	0.4611	0.007
Mdr2	0.5812	0.0004
Cyp8b1	0.6047	0.0002
Cyp7a1	0.6495	0.00004
Ntcp	0.6510	0.00004
FXR (in liver)	0.6944	0.000007
Bacteria in terminal ileum and large intestinal contents		
* Lactobacillus murinus*	−0.9165	0.00003
* Ruminococcus gnavus*	−0.8870	0.0001
* Ruminococcus schinkii*	−0.8574	0.0004
* Lactobacillus acidophilus*	−0.8278	0.0009
* Eubacterium desmolans*	−0.7687	0.003
* Lactococcus lactis*	−0.6209	0.031
* Lactobacillus reuteri*	−0.5617	0.057

## Discussion

Although BBR is used as an herbal medicine and supplement for many ailments, including glucose and lipid metabolism disorders, knowledge about the dose-response of BBR on BAs (which regulate energy homeostasis) and gut flora (which have roles in BA metabolism) in mice are unknown. In the present study, effects of various doses of BBR on BA concentrations in liver and serum, mRNA expression of BA-related genes, as well as the amount of 32 of the most abundant interestinal bacterial species in control mice were examined [30, 34].

Total BAs, including T-conjugated and unconjugated BAs, were not significantly altered in liver, but a change in the serum concentration of BAs was observed in mice treated with BBR. More specifically, unconjugated BAs increased about 90 % in serum of mice treated with 10 and 30 mg/kg BBR, and there was an increase in primary BAs (βMCA, TβMCA and TUDCA) and a decrease in secondary BAs (DCA, LCA and the T-conjugates). With the highest dose of BBR, there was a marked decrease in serum BA concentrations. The expression of BA-synthesis enzymes (Cyp7a1 and 8b1) and uptake transporters (Ntcp) increased 39–400 % in livers of mice treated with higher doses of BBR, whereas nuclear receptors and efflux transporters were not dramatically changed. In addition, *Bacteroides* were exclusively enriched in the terminal ileum and large bowel of mice treated with the higher doses of BBR. Thus, this study shows that BBR has effects on modulating gut microbiota and host BA metabolism.

There are similarities of mice raised in germ-free environment and mice fed BBR. For example, the total BAs in liver were not altered in mice treated with 300 mg/kg BBR (Fig. 2a) or housed in a germ-free environment; however, the total BAs in serum (Fig. 2b) decreased markedly in both experimental groups [14]. As for individual BAs, an increase in βMCA and TβMCA but a decrease in secondary BAs was the major phenotype in liver of both mice given 300 mg/kg BBR (Fig. 3) or housed in a germ-free environment. However, in serum, TβMCA increased markedly in germ-free mice [14] but did not significantly change in mice given 300 mg/kg BBR (Fig. 4a). In addition, the general expression pattern of BA-related genes in liver of 300 mg/kg BBR-treated mice, including increased rate-limiting synthetic enzyme (Fig. 5a) and uptake transporters (Fig. 5c), as well as decreased efflux transporters (Fig. 5d) resembled that in livers of germ-free mice, but in ileum, the suppression of Fgf15 in germ-free mice was not reproduced (Fig. 6) in the 300 mg/kg BBR-treated mice [14].

The expression of the Cyp7a1, the rate-limiting enzyme in the synthetic pathway (Fig. 5a), and Cyp8b1, the 12α-hydroxylase responsible for the synthesis of CA, as well as BA-uptake transporter Ntcp increased in livers of mice treated with the higher doses of BBR (Fig. 5c), but other genes, including the nuclear receptors and efflux transporters, were not altered (Fig. 5d). The expression of FXR increased 63 % in livers of mice treated with 300 mg/kg BBR (Fig. 5b), however, neither the expression of SHP increased nor the BA synthesis enzymes decreased. But in fact, Cyp7a1 and 8b1 were increased, while the FXR-Fgf15 pathway was down-regulated by BBR. Therefore, the decrease in the inhibition of FXR-Fgf15 pathway is likely the reason for the increase of BA synthesis in liver. The changes of individual BAs in the intestine are likely responsible for the decrease in FXR activation in the intestine, as it has been reported that some BAs are antagonists (αMCA, βMCA and their T-conjugates as well as UDCA) and agonists (CDCA, TCDCA, TCA, TDCA, DCA and CA) of the FXR-Fgf15 pathways [14, 16–18]. The most obvious change of the ratio for αMCA + βMCA + TαMCA + TβMCA + UDCA/CDCA + TCDCA + TCA + TDCA + DCA + CA exhibited at 30 mg/kg group. Interestingly, this group also showed the most dramatic decrease of Fgf15 (Fig. 6), which means the FXR signaling activated by BAs in the intestine decreased. This was consistent with the results observed in germ-free mice [14].

BBR is reported to be a broad-spectrum antibiotic, which in this study enriched *Bacteroides* and decreased *Ruminococcus* in the terminal ileum and large bowel (Fig. 7). The present results are consistent with previous work in mice treated with normal chow and a high-fat diet co-administrated with 100 mg/kg BBR [26]. *Bacteroides*, which deconjugates T-conjugated BAs [35], were enriched by BBR. In the current study, this may result in rapid deconjugation of T-conjugated BAs in the intestine, and account for the trend of decreasing TαMCA and TβMCA concentrations in serum of mice treated with the higher BBR doses, leading to decreased amounts of these BAs in the serum. Therefore, the increase of *Bacteroides* might be the reason of the difference of TβMCA in serum between germ-free mice and BBR treatment. However, the possibility that antibiotics might have a direct effect on BA metabolism should not be excluded. Further studies by experimental modulation of the bacteria in the intestine may help to directly prove the association of gut flora changes and the alteration of BA composition and quantity in mice treated with BBR.

Some of the previously reported pharmacological effects of BBR might be related to the change in amount and composition of BAs. In mice treated with higher doses of BBR, the quantity of βMCA and its conjugates were elevated. Moreover, although the increase of UDCA was not statistically significant, the relative abundance of UDCA might increase in parallel with the decrease of other BAs in liver and serum (Figs. 3 and 4). The increase of UDCA, βMCA and their conjugates might be cytoprotective by lowering intracellular TCDCA, which is thought to be cytotoxic due to its hydrophilic nature [36, 37]. βMCA and (T)UDCA also protect against drug-induced cholestasis, possibly by inducing a signaling cascade by activating protein kinase C (PKC) [38], or blocking DCA-induced nuclear factor-kappaB (NF-kappaB) and activator protein-1 (AP-1) activity, as reported in colorectal HCT116 cells, which corresponds to the effects of BBR [21, 39–41]. BBR decreased secondary BAs such as DCA and LCA, which might decrease the hydrophobicity and toxicity of the BA pool [8]. Increased DCA can induce nitric oxide mediated DNA damage [42], and treatment of LCA or its conjugate to animals causes intrahepatic cholestasis [43, 44]. In addition, serum levels of DCA in colon cancer patients have been shown to be consistently higher than that in healthy subjects [45, 46]. In the present study, BBR treatment decreased DCA, LCA, and their conjugates in liver and serum of mice (Figs. 3 and 4), which might relate to the potential of BBR to decrease liver cancer [21]. BAs with two hydroxyl groups (CDCA and DCA) induce fluid secretion, increase mucosal permeability, and produce mucosal damage [47]; thus, a decrease of DCA by BBR treatment may contribute to its anti-diarrheal effect. In addition, BAs have metabolic actions in the body resembling those of hormones by acting through TGR5, and the potency of BAs to activate TGR5 has been reported to be LCA > DCA > CDCA > CA [30]. BBR is an antibiotic, which would decrease secondary BAs in mice and the corresponding composition of BAs might negatively regulate TGR5 in BBR-treated mice. However, BBR has been reported to exhibit similar effects to TGR5 activation [48, 49]. Therefore, further studies are required to quantify the expression and activity of TGR5 in mice treated with BBR. Additionally, determining the effect of BA feeding combined with BBR treatment, and the influence of BBR on genetically modified mouse models might provide us with clues to the underlying relationship between BBR and BAs.

## Conclusion

The results of the present study showed that various doses of BBR have effects on BA metabolism and signaling pathways as well as gut flora, which will provide guidance for further studies to determine the mechanisms of BBR effects.

## Additional file

Additional file 1: Table S1. RT-PCR primers for related genes. (DOCX 15 kb)

## Abbreviations

BA: Bile acid
BBR: Berberine
Bsep: Bile salt export pump
CA: Cholic acid
CAR: Constitutive androstane receptor
CDCA: Chenodeoxycholic acid
Cyp: Cytochrome P450
DCA: Deoxycholic acid
EHC: Enterohepatic circulation
Fgf: Fibroblast growth factor
Fgfr4: FGF receptor 4
FXR: Farnesoid X receptor
G: Glycine
HDCA: Hyodeoxycholic acid
LCA: Lithocholic acid
LRH-1: Liver receptor homolog-1
MCA: Muricholic acids
MDCA: Murideoxycholic acid
Mdr: Multiple drug resistance
mRNA: Messenger RNA
Mrp: Multidrug resistance-associated protein
NF-kappaB: Nuclear factor-kappaB
Ntcp: Na^+^-dependent taurocholate cotransporting peptide
Oatp: Organic anion–transporting polypeptide
Ost: Organic solute transporter
PK: Protein kinase
SHP: Small heterodimer partner
T: Taurine
TGR5: Transmembrane G-protein-coupled receptor 5
UDCA: Ursodeoxycholic acid
UPLC-MS: Ultraperformance liquid chromatography–mass spectrometry
