# Supplementary material for: Dose-response effect of berberine on bile acid profile and gut microbiota in mice
Source: BMC Complement Altern Med. 2016 Oct 18;16:394. doi: 10.1186/s12906-016-1367-7 (PMC5070223; doi:10.1186/s12906-016-1367-7)
Supplement: Additional file 1: Table S1. — RT-PCR primers for related genes. (DOCX 15 kb) [file 12906_2016_1367_MOESM1_ESM.docx]

**Additional file 1: Table S1 RT-PCR primers for related genes**

| Gene | Gene bank No. | Position | Forward | Reverse |
| --- | --- | --- | --- | --- |
| Synthetic enzyme |  |  |  |  |
| Cyp7a1 | NM_007824 | 165-283 | caacgggttgattccatacc | atttccccatcagtttgcag |
| Cyp8b1 | NM_010012 | 730-835 | agttgcagcgtctcttccat | ccttgctccctcagaaactg |
| Cyp27a1 | NM_024264 | 646-767 | gagagtgaatcaggggacca | tcaggaatggagggtttcag |
| Cyp7b1 | NM_007825 | 1104-1220 | cctgcagtcaacaggtcaaa | gagcacagcctcagaacctc |
| Regulator |  |  |  |  |
| FxR | NM_009108 | 357-503 | tgggtaccagggagagactg | gtgagcgcgttgtagtggta |
| SHP | NM_011850 | 699-820 | ctcatggcctctaccctcaa | ggtcacctcagcaaaagcat |
| Fgfr4 | NM_008011 | 1204-1350 | ctgccagaggaagacctcac | gtagtggccacggatgactt |
| Uptake transporter |  |  |  |  |
| Ntcp | NM_011387 | 594-622 | ggtgccctacaaaggcatta | acagccacagagagggagaa |
| Oatp1b2 | NM_020495 | 204-290 | caaactcagcatccaagcaa | ggctgccaaaaatatcctga |
| Efflux transporter (canalicular) |  |  |  |  |
| Bsep | NM_021022 | 2308-2411 | ggacaatgatgtgcttgtgg | cacacaaagcccctaccagt |
| Mrp2 | AF227274 | 2685-2760 | tgcagcttccttgaccatga | cctgctgccggacctagag |
| Mdr2 | U46840 | 737-846 | tggccgatgtgtgtgagtaca | tgcctggcaccaaaaggt |
| Bcrp | NM_011920 | 391-524 | tgaggcctgacagttctcct | atcctaggaaggccgttgtt |
| Efflux transporter (basolateral) |  |  |  |  |
| Ostß | NM_178933 | 226-369 | atcctggcaaacagaaatcg | ggccaagtctggtttctctg |
| Mrp3 | NM_029600 | 3120-3222 | tggtcatgctgtcagctttc | aaggactgaggggaacgaat |
| Mrp4 | BC150822 | 1257-1395 | gcaaagcccatgtaccatct | accacggctaacaactcacc |
| Ileum genes |  |  |  |  |
| Fgf15 | NM_008003 | 430-637 | gaagacgattgccatcaagg | gtcctggagctgttctctgg |
| Ostα | NM_145932 | 49-196 | ttgtgatcaaccgcatttgt | ctcctcaagcctccagtgtc |
| Asbt | NM_011388 | 835-951 | tggaatgcagaacactcagc | gcaaagacgagctggaaaac |
